# Oxidative Stress Markers in Chronic Kidney Disease with Emphasis on Diabetic Nephropathy

**DOI:** 10.3390/antiox9100925

**Published:** 2020-09-27

**Authors:** Nina Vodošek Hojs, Sebastjan Bevc, Robert Ekart, Radovan Hojs

**Affiliations:** 1 Department of Nephrology, Clinic for Internal Medicine, University Medical Centre Maribor, Ljubljanska 5, 2000 Maribor, Slovenia; nina.hojs1@gmail.com (N.V.H.); sebastjan.bevc@gmail.com (S.B.); 2 Faculty of Medicine, University of Maribor, Taborska 8, 2000 Maribor, Slovenia; robert.ekart2@guest.arnes.si; 3 Department of Dialysis, Clinic for Internal Medicine, University Medical Centre Maribor, Ljubljanska 5, 2000 Maribor, Slovenia

**Keywords:** oxidative stress, antioxidants, biomarkers, diabetic nephropathy, chronic kidney disease

## Abstract

Diabetes prevalence is increasing worldwide, especially through the increase of type 2 diabetes. Diabetic nephropathy occurs in up to 40% of diabetic patients and is the leading cause of end-stage renal disease. Various factors affect the development and progression of diabetic nephropathy. Hyperglycaemia increases free radical production, resulting in oxidative stress, which plays an important role in the pathogenesis of diabetic nephropathy. Free radicals have a short half-life and are difficult to measure. In contrast, oxidation products, including lipid peroxidation, protein oxidation, and nucleic acid oxidation, have longer lifetimes and are used to evaluate oxidative stress. In recent years, different oxidative stress biomarkers associated with diabetic nephropathy have been found. This review summarises current evidence of oxidative stress biomarkers in patients with diabetic nephropathy. Although some of them are promising, they cannot replace currently used clinical biomarkers (eGFR, proteinuria) in the development and progression of diabetic nephropathy.

## 1. Introduction

Chronic kidney disease (CKD) is a common and serious disease that affects 8 to 16% of the global population [1]. Management of CKD is costly and it presents a significant challenge for societies and health care systems [2]. In 2016, CKD was the 16^th^ leading cause of years of life lost worldwide, mainly due to cardiovascular diseases and infections, and is expected to rise to 5th place by 2040 [3]. The increasing prevalence of CKD is associated with the increase in patients with diabetes and hypertension [4,5,6]. In 2019, 463 million people had diabetes and the International Diabetes Federation estimates that there will be 700 million adults with diabetes by 2045 [6]. Diabetes prevalence is increasing, especially through an increase in type 2 diabetes [6]. Diabetes is the main cause of CKD in many developed countries and is quickly becoming the leading cause in developing countries [4]. Diabetic nephropathy (DN) occurs in up to 40% of type 1 or type 2 diabetic patients [7]. Other frequent causes of CKD are hypertension, glomerulonephritides, etc.; in many cases, the cause of CKD is unknown [2,8]. 

Oxidative stress contributes to many pathological conditions. It is involved in the onset and/or progression of cancer, atherosclerosis, neurological disorders, cardiovascular diseases, pulmonary diseases, and diabetes [9,10,11,12,13,14,15]. Diabetes mellitus is a chronic disease with either a lack of insulin production or, more commonly, resistance to insulin, leading to hyperglycaemia. Hyperglycaemia increases free radical production, leading to oxidative stress [16]. Experimental and clinical studies suggest an association between hyperglycaemia, oxidative stress, and diabetic complications [16,17,18,19]. Oxidative stress plays an important role in the pathogenesis of DN and its progression to end-stage renal disease (ESRD) [16,20,21,22].

In the current review, we will present the role of oxidative stress in patients with diabetes and CKD. Our emphasis will be presenting the oxidative stress markers in the development and progression of diabetic nephropathy used in clinical studies.

## 2. Oxidative Stress

Oxidative stress is a state of imbalance between oxidants and antioxidants [23]. It is dependent on the production and accumulation of oxidant radicals in cells and tissues and the ability of a biological system to detoxify these reactive products [24]. Oxidant compounds (reactive oxygen species (ROS), reactive nitrogen species (RNS)) are products of normal cellular metabolism. We can divide them into free radicals and nonradicals [25]. Free radicals have one or more unpaired electrons and are therefore highly reactive [23,25]. Examples of the most important ROS and RNS of physiological significance are superoxide anion (O_2_^•−^), hydroxyl radical (^•^OH), nitric oxide radical (NO^•^), and nitrogen dioxide radical (NO_2_^•^) [9]. When two free radicals share their unpaired electrons, nonradicals are formed. More often, free radicals attack nonradical molecules and a new radical molecule is formed, triggering a chain reaction [23]. Examples of nonradical oxidants are hydrogen peroxide (H_2_O_2_), ozone (O_3_), singlet oxygen (^1^O_2_), hypochlorous acid (HOCl), nitrous acid (HNO_2_), dinitrogen trioxide (N_2_O_3_), peroxynitrite (ONOO^−^), and lipid peroxides [23]. 

At low to moderate concentrations, ROS and RNS act as secondary messengers and regulate intracellular signal transduction pathways regulating cell growth and differentiation, mitogenic responses, extracellular matrix production and breakdown, apoptosis, oxygen sensing, and inflammation [26,27]. ROS and RNS act as part of the immune defence system [27]. At high concentrations, they produce unwanted modifications to lipids, proteins, DNA, etc. [25]. ROS are very unstable with short half-lives (only seconds) and are therefore difficult to measure [23]. In contrast, oxidation products have longer lifetimes (from hours to weeks) and are used to assess the redox state [23,28]. The most important markers of oxidative stress are presented in Table 1.

### 2.1. Sources of Oxidative Stress

Normal aerobic metabolism is a major source of ROS; the most important in ROS generation are mitochondrial nicotinamide adenine dinucleotide phosphate (NADPH) oxidase (NOX), xanthine oxidase (XO), myeloperoxidase (MPO), and endothelium nitric oxide synthase (eNOS) [23,25,29]. Other enzyme sources are prostaglandin synthase, lipoxygenase, and flavoprotein dehydrogenase [23,25,29]. The main exogenous sources of oxidative stress are cigarette smoke, environmental pollution, heavy metals (Cd, Hg, Pb, Fe, and As), drugs (gentamycin, bleomycin, etc.), alcohol, chemical solvents, and radiation [23,24,25,29].

### 2.2. Antioxidants

The human body has defence mechanism that counterbalances the effects of oxidants: the antioxidants. They can be divided into enzymatic and nonenzymatic antioxidants. The major enzymatic antioxidants are superoxide dismutase (SOD), catalase, glutathione peroxidase (GSH-Px), haem oxygenase-1 (HO-1), and thioredoxin [23,24,25,30,31,32,33,34]. The major nonenzymatic antioxidants are glutathione (GSH), vitamins (vitamins C and E), and β-carotene [23,24,25,35,36,37]. They are low-molecular-weight compounds and are found in the plasma, extracellular fluids, intracellular fluids, lipoproteins, and membranes [9]. An important endogenous antioxidant with good antioxidant capacity is serum albumin [23,38]. There are also several exogenous antioxidant molecules (polyphenols, flavonoids) which are mainly introduced by the diet or by nutritional supplementation [23,25,39]. Enzymatic and nonenzymatic antioxidants are presented in Table 1.

## 3. Oxidative Stress in CKD

**Figure 1:**
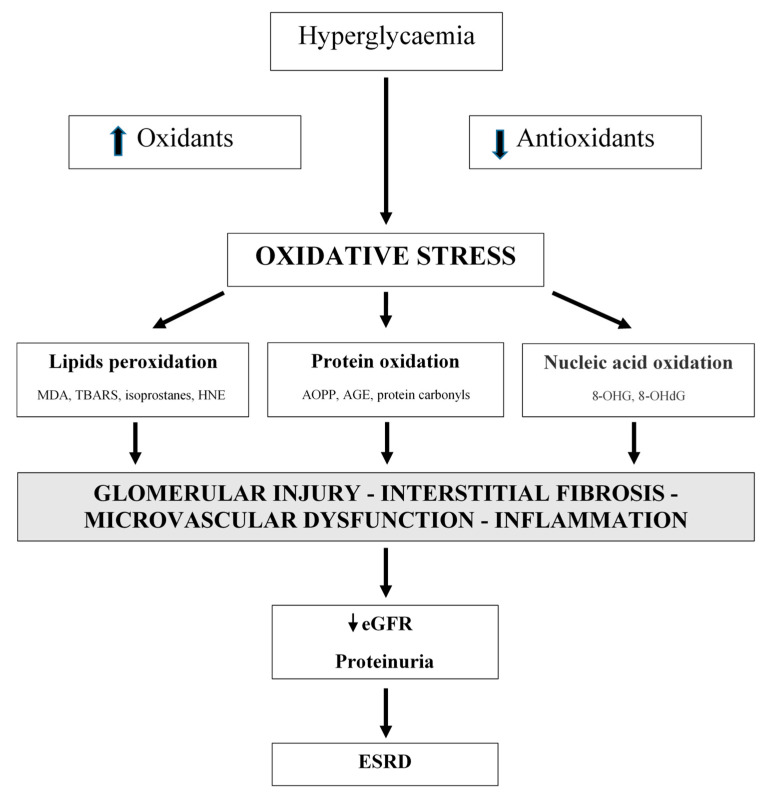
Oxidative stress is a significant factor in the development of diabetic nephropathy. Oxidative stress is associated with metabolic changes and alterations in renal hemodynamic. MDA: malondialdehyde; TBARS: thiobarbituric acid reactive substances; HNE: 4-hydroxynonenal; AOPP: advanced oxidation protein products; AGE: advanced glycation end products; 8-OHG: 8-hydroxyguanosine; 8-OHdG: 8-hydroxy-2′-deoxyguanosine; eGFR: estimated glomerular filtration rate; ESRD: end-stage renal disease.

Oxidative stress is not only an important factor in the development of type 1 and type 2 diabetes, but it also has a significant role in the development of diabetic complications, including DN [11,14,16,17,18,19,20,21,22,40,41,42]. Oxidative stress is linked with metabolic changes and alterations in renal haemodynamics. Both mechanisms have adverse synergistic effects [40]. Oxidative stress is directly linked to podocyte damage, proteinuria, and tubulointerstitial fibrosis [43]. Additionally, vascular oxidative stress has an important role in CKD progression (Figure 1) [43,44,45,46].

### 3.1. Oxidative Stress and Glomerular Injury

Podocytes are vulnerable to oxidative damage [43]. Mature podocytes are highly differentiated cells and respond to injury with detachment from the glomerular basement membrane, dedifferentiation, autophagy, and apoptosis [47]. An important consequence of podocyte injury is proteinuria, which is a well-known marker of kidney damage and is associated with CKD progression [47,48]. Proteinuria is an important factor in inducing mesangial and tubular toxicity and is involved in local and systemic inflammatory pathways [48,49]. 

In early studies, it was shown that puromycin aminonucleoside, a podocyte toxin, induced glomerular injury in rats through ROS [43,50,51]. In these studies, antioxidants also provided protection against the changes in podocytes [51]. Later, ROS-mediated DNA damage was also shown [52]. Podocyte injury and dysfunctional glomerular filtration barrier is important in the process of focal segmental glomerular sclerosis (FSGS). The development and progression of FSGS is associated with transforming growth factor beta (TGF-β) activation in podocytes [53]. TGF-β is involved in crosstalk between podocytes and the glomerular endothelium [54]. TGF-β promotes synthesis of endothelin precursors in podocytes and expression of endothelin receptors. The binding of endothelin with its receptors suppresses mitochondrial function and induces oxidative stress in the glomerular endothelium [54]. Mitochondrial oxidative DNA damage was evident before podocyte injury [54]. 

Other oxidative stress markers are advanced oxidation protein products (AOPPs). They are dityrosine-containing products of plasma proteins [43]. Higher AOPP levels were found in patients with CKD compared to controls [55,56]. Podocyte injury, proteinuria, and glomerulosclerosis were associated with AOPPs through a NOX-dependent mechanism [57]. In normal rats, chronic administration of AOPPs increased proteinuria and urinary 8-hydroxydeoxyguanosine (8-OHdG) excretion. On the other hand, chronic inhibition of NOX by apocynin prevented podocyte apoptosis and decreased proteinuria in these rats [57]. AOPPs interacted with the receptor of advanced glycation end products (RAGE) on podocytes [58]. Additionally, blocking RAGE by anti-RAGE immunoglobulin G or its silencing by siRNA significantly protected podocytes from AOPP-induced apoptosis and ameliorated proteinuria in AOPP-challenged mice [58]. AOPPs are involved in the activation of Wnt/β-catenin signalling. Wnts are a family of secretory proteins that induce a series of signals which results in the phosphorylation of β-catenin [59]. After activation, β-catenin enters the nucleus and promotes the transcription of Wnt target genes [59]. Wnt/β-catenin signalling is silent in normal adults. AOPPs induce NOX activation via plasma membrane receptor RAGE, which promotes the activation of the nuclear factor kappa B (NF-κB) transcription factor. The NF-κB transcription factor leads to the induction of Wnt ligands, such as Wnt1 and Wnt7a, and the activation of β-catenin [60]. Accumulating evidence suggests that Wnt/β-catenin has an important role in oxidative stress-induced podocyte damage and proteinuria [60]. Recently, it was demonstrated that a blockade of Wnt signalling preserves podocyte integrity and ameliorates proteinuria [60]. According to the mentioned data, targeting Wnt/β-catenin could be a new therapeutic modality for proteinuric CKD [60].

In the middle-aged general population, a marker of oxidative DNA damage, urinary 8-hydroxyguanosine (8-OHG) excretion, was independently associated with incident low-grade albuminuria during almost 6 years of follow-up [61].

Additionally, oxidative stress is also associated with progressive renal failure. Finnish-type congenital nephrotic syndrome (NPHS1) is a rare genetic kidney disease caused by mutations in the NPHS1 gene, which codes for the podocyte protein nephrin [62]. The disease is characterised by heavy proteinuria and hypoproteinaemia from birth [62]. In nephrectomised kidneys from children with NPHS1, interstitial expression of MPO was demonstrated [62]. This enzyme generates hypoclorous acid (HOCl), which causes irreversible tissue damage [62]. The concentration of free GSH in the cortex of the NPHS1 kidneys, which is a major antioxidant, was extremely low as compared to controls [62]. All these findings support the fact that proteinuric kidneys are under heavy oxidative stress. 

In proteinuric CKD, tubulointerstitial injury with subsequent progressive loss of renal function is common. During urinary albumin endocytosis in the proximal tubule, protein kinase C-dependent NOX-mediated ROS generation is induced and this is responsible for enhanced NF-κB activity and the induction of NF-κB-dependent pathways of interstitial inflammation [63,64].

Less is known about the role of antioxidants in proteinuric CKD. Enzyme superoxide dismutase (SOD) protects the kidney from superoxide. Downregulation of cytosolic CuZn-SOD (SOD1) and extracellular CuZn-SOD (SOD3), but not mitochondrial Mn-SOD (SOD2), was observed in the kidney of KK/Ta-Akita mice that exhibit progressive DN [65]. In this study, no change in renal SOD expression in DN-resistant C57BL/6-Akita mice was observed [65]. In another study, a murine model of adriamycin-induced nephropathy was used. Levels of SOD3 diminished throughout the course of disease progression [66]. Interestingly, similar to findings in mice, a decrease in SOD3 in human CKD biopsy samples was found [66]. The authors concluded that SOD3 protects against proteinuric renal injury in vivo. It offers protection through the inhibition of NOX upregulation and downregulation of pathologic β-catenin signalling [66]. 

### 3.2. Oxidative Stress and Interstitial Fibrosis

Disregarding the initial injury, renal fibrosis is the common final pathway leading to ESRD, and the degree of fibrosis or fibroblast number are robust pathologic markers of progression [67]. Tubulointerstitial fibrosis includes the deposition of interstitial matrix with inflammatory cells, tubular cell loss, fibroblast accumulation, and rarefaction of the peritubular microvasculature [67]. Renal scarring is a result of complex interactions of molecular pathways, growth factors, cytokines, and cells [68,69,70,71].

Fibroblasts/myofibroblasts are most responsible for interstitial matrix accumulation and subsequent structural changes [72]. Collagen-producing myofibroblasts in the kidney can be derived from resident fibroblasts, pericytes, perivascular adventitial, epithelial, and/or endothelial sources [72]. Regardless of the origin of the cells, TGF-β1 is the main molecule responsible for myofibroblast activation with the expression of α-smooth muscle actin (α-SMA), which gives the myofibroblasts their contractility [72,73,74]. TGF-β1 increases the activity of NOX and expression of NOX2 and NOX4, homologues of the NOX family, indicating that this growth factor induces the production of ROS [74]. NOX2 and NOX4 have an important role in the conversion of fibroblasts to myofibroblasts [72,74]. It was shown that inhibition of NOX4 inhibited TGF-β-induced stimulation of NOX activity and reduced α-SMA expression [74]. Additionally, inhibition of TGF-β receptor type I reduced TGF-β-enhanced NOX activity and decreased expression of NOX4 and α-SMA [74]. 

As was shown, NOX synthesises ROS that are involved in fibrosis progression. On the other hand, their effect on renal disease progression is not well understood. In the model of chronic renal injury due to unilateral urinary obstruction, leading to renal fibrosis, wild-type and NOX4-deficient mice were used [75]. In the NOX4-deficient mice, more interstitial fibrosis was found in the obstructed kidney compared to the wild-type mice [75]. More TGF-β1-mediated tubular apoptosis, reduced expression of hypoxia-inducible factor-1α, and vascular endothelial growth factor was also found in the obstructed kidneys of the NOX4-deficient mice [75]. It was shown that the absence of NOX4 increases interstitial kidney fibrosis, independent of NOX2. [75]. NOX4 deficiency increased fibrosis due to enhanced tubular cell apoptosis, decreased microvascularisation, and enhanced oxidative stress [75]. The NOX4-mediated protection might be a consequence of Nrf2 pathway upregulation [76]. The Nrf2/Keap1 system controls the expression of antioxidant genes [76]. Furthermore, Nrf2 plays a protective role in CKD animal models, including DN [77,78].

Uraemic toxins are also involved in the progression of CKD. In the last decade, indoxyl sulphate (IS) and p-cresyl sulphate (PCS), which accumulate with CKD progression, have appeared as key nephrotoxins [79,80]. IS and PCS enhance ROS production in renal tubular cells, which activate the NF-kB pathway, resulting in both oxidative stress and inflammation [80,81]. These mechanisms have been confirmed in studies showing that fibrosis of renal tubules and oxidative stress are significantly enhanced after toxin administration and suppressed after IS reduction [80,81,82]. Additionally, it was shown that antioxidant treatment dose-dependently inhibits the fibrotic and oxidative effects of IS and PCS [83,84].

Recently, it was demonstrated that oxidative stress and autophagy are involved in kidney health and disease [85]. Autophagy is a crucial cellular homeostatic process that cells use to degrade and recycle cellular proteins and remove damaged organelles. It involves the formation of double membrane-bound vesicles called autophagosomes, which later fuse with lysosomes [86]. Basal levels of redox signalling and autophagy signalling are necessary to maintain cellular homeostasis. Under distinct circumstances, changes in autophagic flux have been shown to regulate ROS formation and redox signalling [85]. It is also suggested that ROS and RNS induce autophagy and vice versa [85,87].

### 3.3. Oxidative Stress and Microvascular Dysfunction

The endothelium is a fundamental layer in the arterial wall and is essential for the regulation and maintenance of normal renal function [43,44]. Oxidative stress is related to endothelial dysfunction and plays a critical role in CKD progression [44,45,88]. The endothelium secretes nitric oxide (NO), which is produced from arginine by the enzyme NOS [88]. NO is involved in several biological processes, including vasodilatation in smooth muscle cells, inflammation, and immune responses [88]. NOS is expressed as various isoforms: endothelial NOS (eNOS), neuronal NOS (nNOS), inducible NOS (iNOS), and constitutive NOS (cNOS); all have been isolated from the kidney [43,88,89]. The cNOS is expressed in the vessels, glomeruli, and tubules, iNOS is expressed in vascular smooth muscle cells and the mesangium, and eNOS is associated with the vascular endothelium [45,88,90]. Low levels of NO in the endothelium induce the expression of antioxidative genes and protect renal endothelial and mesangial cells from apoptosis and fibrosis but, on the other hand, increased levels of ROS reduce the production of NO via inhibition and/or uncoupling of NOS enzymes [45,88,89,90]. The NO production in the kidney can be blocked by NOS inhibition with asymmetric dimethylarginine (ADMA). ADMA is a natural product formed by the methylation of arginine which accumulates in the plasma of CKD patients in the early stages of CKD [45,89]. The decrease in NO leads to an increase in vascular resistance [89]. Additionally, it was shown in patients with CKD stages 1-5 that levels of serum ADMA and oxidative stress markers (plasma malondialdehyde (MDA), erythrocyte SOD, and GSH-Px) were directly associated with CKD stages [45]. It was shown that the glomerular filtration rate correlated negatively with plasma MDA and ADMA levels and positively with erythrocyte SOD and GSH-Px [45]. Patients with CKD, compared to a control group of healthy subjects, had higher levels of MDA and ADMA and lower levels of erythrocyte SOD and GSH-Px [45]. Furthermore, it was shown that levels of oxidative stress markers and ADMA are independently associated with endothelial function [45].

Autoregulation is important in maintaining renal blood flow, glomerular filtration rate, and tubular fluid flow over a wide range of perfusion pressures. It is dependent on afferent arteriole contraction followed by a tubuloglomerular feedback [91,92]. Impairment of renal autoregulation is associated with CKD progression. In experimental studies, it was documented that ROS mediate myogenic responses of afferent arterioles in CKD models [93]. It was also shown that NOX2 plays an important role in regulating tone and reactivity of afferent arterioles, also in response to angiotensin II (ANG II) and/or adenosine [94]. NOX2-derived ROS scavenges NO, causing subsequent NO deficiency [94]. It was demonstrated that an increase in perfusion pressure increases superoxide (O_2_^•−^) in afferent arterioles in normal mice or mice with a genetic deletion of SOD and is involved in the myogenic contractions of afferent arterioles [95,96]. H_2_O_2_ impaired autoregulation of afferent arterioles in five out of six nephrectomised mice [92,95,96]. 

### 3.4. Oxidative Stress and Chronic Inflammation

Oxidative stress and inflammation, as well as their interaction, have an important role in the pathogenesis and progression of CKD [97]. Both promote renal injury through damage of molecular components [98]. The primary pathological mechanism that links oxidative stress, inflammation, and CKD progression includes an initial injury to the kidney by intra- and extracellular oxygen-derived radicals and the resultant inflammation [98]. In recent years, some important review papers have been published showing the importance of inflammation in the pathogenesis and progression of CKD [16,20,23,43,99,100,101,102,103]. More details about the role of inflammation in CKD is beyond the scope of this review.

## 4. Biomarkers of Oxidative Stress in Patients with CKD

ROS are highly reactive and unstable compounds with short half-lives of only seconds. Using them as clinical biomarkers of oxidative stress is difficult or even impossible. ROS produce unwanted modifications to lipids, proteins, DNA, etc. These oxidation products have longer lifetimes and, together with antioxidants, are used to assess the redox state.

### 4.1. Lipid Peroxidation

Oxidation of polyunsaturated fatty acids (linoleic acid, arachidonic acid, etc.) by free radicals is known as lipid peroxidation and it can cause major tissue damage [104]. Lipids are the main component of cellular membranes and peroxidation alters their properties and consequently affects their function [105]. The most frequently studied lipid peroxidation markers are MDA, 4-hydroxynonenal (HNE), thiobarbituric acid reactive substances (TBARSs), and isoprostanes such as 8-iso-prostaglandin F_2*α*_ (8-iso-PGF_2*α*_) [106,107,108]. 

MDA is formed through lipid peroxidation and during prostaglandin and thromboxane synthesis [105]. It can attack macromolecules, leading to alterations in their functions [105]. In several studies, higher serum MDA levels were found in CKD patients compared to healthy control subjects [109,110,111]. MDA correlated negatively with the glomerular filtration rate and was significantly different among CKD patients with stages 2, 3, 4, and 5 [31]. Higher levels of serum MDA were also found in haemodialysis patients [31,112]. In haemodialysis patients, the value of serum MDA is limited because it is a water-soluble low-molecular-weight product and could be removed by haemodialysis [105]. Serum MDA levels in transplant patients were significantly lower than in dialysis patients [113]. Furthermore, serum MDA decreased after kidney transplantation [114].

TBARSs are a nonspecific marker of lipid peroxidation, therefore, serum MDA or F2-isoprostanes are preferred alternatives. It was shown that the production of TBARSs was higher in advanced CKD stages and in haemodialysis patients [115]. 

F2-isoprostanes are lipid peroxidation products with a prostaglandin-like structure formed by the nonenzymatic oxidation of arachidonic acid, and could be detected in serum and urine samples [105]. The values of plasma F2-isoprostanes were higher in CKD patients and in ESRD patients (both haemodialysis and peritoneal dialysis) compared to control subjects [116,117,118]. F2-isoprostanes increased significantly as the CKD stage advanced and were inversely related to the glomerular filtration rate [119]. Moreover, F2-isoprostanes significantly decreased after kidney transplantation [120].

### 4.2. Protein Oxidation

Protein oxidation is a covalent modification induced directly by ROS and/or RNS or indirectly by reaction with secondary products of oxygen stress [107]. Oxidative modifications lead to changes in protein properties and the consequences are loss of enzymatic activity, altered cellular functions, interference with the creation of membrane potentials, and changes in the type and level of cellular proteins [121,122].

Protein tyrosine nitration is mediated by RNS such as peroxynitrite (ONOO^−^) and nitrogendioxide (NO_2_) and results in structural and functional changes, leading to altered cell homeostasis [123]. It was shown that nitrotyrosine was higher in haemodialysis patients compared to controls [124]. It is important to note that methods of detecting nitrotyrosine are quite costly and impractical for daily screening and analysis [123].

AOPPs and AGEs are markers of protein oxidation and proinflammatory mediators [107]. AOPPs are increased in CKD and ESRD patients and higher levels were found in dialysis patients [125]. All of them showed increased AOPP levels in comparison to age-matched controls [125]. It is important to note that AOPP levels are overestimated in patients with hypertriglyceridaemia [125]. 

AGEs, such as pentosidine, were increased in CKD and ESRD patients [126]. In nondialysis CKD patients, pentosidine was associated inversely with the glomerular filtration rate [126]. 

Protein carbonylation is the oxidation of proteins that can be promoted by ROS, and protein carbonyls are used as markers of oxidative stress. Plasma protein carbonyl levels were higher in CKD and haemodialysis patients compared to normal volunteers [127]. In this study, no significant difference in the plasma protein carbonyl group concentration between CKD patients and chronic haemodialysis patients was found [127]. Results from another study showed that carbonylation of albumin in CKD patients gradually increased during the development of the disease [128]. The carbonylation of albumin was even higher in the plasma of haemodialysis patients, while a comparison of peritoneal dialysis patients with controls found no difference [128]. Protein carbonyls were inversely related to the glomerular filtration rate and a significant reduction in plasma carbonyls after renal transplantation was documented [129].

### 4.3. Nucleic Acid Oxidation

Oxidative damage to DNA includes fragmentation products, single/double-strand breaks, inter/intra-strand cross-links, DNA protein cross-links, and DNA bases damage [107]. Sensitive biomarkers of DNA damage are 8-hydroxyguanosine (8-OHG) and 8-hydroxy-2′-deoxyguanosine (8-OHdG).

8-OHdG levels in peripheral leukocyte DNA were higher in CKD patients compared to healthy controls. The highest values were observed in peritoneal dialysis patients [130]. Furthermore, in nondialysed CKD patients, 8-OHdG levels inversely correlated with renal creatinine clearance [130]. An increased 8-OHdG level in leukocyte DNA was also found in haemodialysis patients [131]. They had the greatest 8-OHdG level, followed by undialysed CKD patients and healthy controls [131]. The 24 h urinary 8-OHdG excretion in patients with proteinuria was significantly higher than in the control subjects [132]. 

### 4.4. Antioxidants

An antioxidant is a substance that delays or inhibits cell damage caused by free radicals [133]. Total antioxidant status is determined by different measurement techniques and the results are difficult to compare across studies [107]. Patients with CKD, including haemodialysis patients, have diminished total antioxidant capacity [134,135]. It was reported that total antioxidant capacity was also lower in peritoneal dialysis patients [136].

The first line of enzymatic antioxidant defence is SOD, which dismutes superoxide hydrogen peroxide and molecular oxygen [107]. Results from studies examining SOD in CKD patients are contradictory and difficult to interpret [107]. Some authors found no significant difference in SOD between CKD patients and controls [88,137], while others found reduced SOD activity in haemodialysis and peritoneal dialysis patients compared to controls [138], or that plasma SOD activity increased in CKD patients with the progression of renal insufficiency [139]. On the other hand, plasma SOD values were lower in CKD patients than controls and the glomerular filtration rate correlated positively with SOD [31]. Erythrocyte SOD levels increased following renal transplantation [140]. The lack of consistency in SOD expression encourages careful interpretation of the results [107]. 

Catalase reduces H_2_O_2_ to water; selenium-containing GSH-Px reduces all organic lipid peroxides and requires GSH as a hydrogen donor [28,107]. Studies related to plasma or erythrocyte catalase and GSH-Px activity are conflicting and the results should be interpreted very carefully [88,135,138,139,141,142,143,144,145]. 

GSH, a tripeptide, is a major nonenzymatic antioxidant found in almost all living cells. It is considered as a biomarker of redox imbalance at the cellular level and its activity fluctuates less than other antioxidants, making it a more stable indicator of antioxidant status [107,137]. Plasma GSH was diminished in many studies, including CKD, haemodialysis, and peritoneal dialysis patients [144,146,147]. Interestingly, GSH concentration measured in erythrocytes showed contradictory results, it may either be decreased, unchanged, or even increased [148,149,150,151,152]. GSH is oxidised to glutathione disulphide (GSSG); GSSG and the GSH/GSSG ratio were used as markers of GSH-related activity in some studies [107,153].

## 5. Biomarkers of Oxidative Stress in Development and Progression of DN

Diabetes is the leading cause of CKD. The exact pathogenesis is complex and oxidative stress has a significant role in the pathogenesis of DN and its progression to ESRD. In recent years, a variety of biomarkers of oxidative stress associated with DN has been found and the most important ones used in clinical studies are presented (Table 2) [152,153,154,155,156,157,158,159,160,161,162,163,164,165,166,167,168,169,170,171,172,173,174,175,176,177,178,179,180,181,182,183,184,185,186].

### 5.1. Lipid Peroxidation

According to lipid peroxidation markers, an increase in MDA or TBARSs in type 2 diabetic patients with and without complications compared to healthy controls is one of the most consistent findings [105]. Furthermore, a significant increase in MDA and TBARSs in type 2 diabetic patients with micro- and macrovascular complications compared to those without was reported [154,155,156]. In these studies, up to 40% of patients with microvascular complications had DN. Unfortunately, patients with DN were not analysed separately. In type 2 diabetic patients with DN, it was shown that MDA was significantly higher in patients with DN compared to patients without DN and healthy controls [157,158,159]. Meanwhile, other studies reported no difference in MDA between patients with or without DN [160,161].

A significant increase in urinary and plasma levels of total F2-isoprostanes was found in type 2 diabetic patients with DN compared to controls [162].

### 5.2. Protein Oxidation

AOPPs were higher in type 2 diabetic patients compared to controls [154,156,163,164]. AOPPs were increased in patients with micro- or macrovascular complications (including DN) compared to those without them [154,156,163,164]. Patients with DN were not analysed separately in these studies. It was documented that diabetic patients with albuminuria had increased AOPP levels compared to those without albuminuria [165].

Plasma AGE levels were higher in type 2 diabetic patients compared to healthy controls and in type 2 diabetic patients with micro- or macrovascular complications (including DN) compared to those without complications [156,159,163,166,167]. In another study, AGEs were significantly higher only in type 2 diabetic patients with chronic renal failure (defined as creatinine ≥ 1.3 mg/dL) compared to patients with normo-, micro-, and macroalbuminuria without renal failure [168]. On the contrary, no difference was found in AGEs when comparing type 2 diabetic patients with or without nephropathy [161]. 

In type 1 diabetic patients, serum levels of AGEs were significantly increased as normal renal status advanced to microalbuminuria, clinical nephropathy, and haemodialysis; serum levels of AGEs positively correlated with urinary albumin excretion [169].

Protein carbonyls were also higher in type 2 diabetic patients compared to healthy controls and in type 2 diabetic patients with micro- or macrovascular complications (including DN) compared to those without complications [154,156,163,164]. Furthermore, increased levels of plasma and lymphocyte carbonyls were found in type 2 diabetic patients with DN compared to healthy controls [162].

### 5.3. Nucleic Acid Oxidation

Increased serum and urinary 8-OHdG in type 2 diabetic patients was documented compared to controls [158,170,171]. Increased serum and plasma 8-OHdG was documented in type 2 diabetic patients with DN compared to diabetic patients without complications [158,172]. It was also found that plasma 8-OHdG levels in diabetic patients with micro- and macroalbuminuria were increased compared to normoalbuminuric patients [161]. Moreover, urinary 8-OHdG levels were significantly higher in patients with microvascular complications, including DN, compared to those without complications [173]. Urinary 8-OHdG levels in type 2 diabetic patients were significantly higher in patients with macroalbuminuria compared to patients with micro- or normoalbuminuria [132,174]. Additionally, urinary 8-OHdG levels were increased in type 2 diabetic patients with micro- and macroalbuminuria compared to patients with normoalbuminuria and healthy controls; 8-OHdG levels were also significantly higher in patients with macroalbuminuria compared to patients with microalbuminuria [175]. In a prospective longitudinal study, patients with higher urinary excretion of 8-OHdG had a significant progression of DN compared to patients with moderate or lower excretion of 8-OHdG [176]. In this study, multivariate logistic regression analysis suggested that urinary 8-OHdG was the strongest predictor of nephropathy among several known risk factors [176]. Interestingly, no significant association between leukocyte 8-OHdG and the development of nephropathy was found [176]. On the other hand, no difference was found in urinary 8-OHdG levels in type 2 diabetic patients with or without DN [170]. Furthermore, an RNA oxidation marker, urinary 8-OHG, was also elevated in type 2 diabetic patients with and without complications compared to age-matched healthy controls [171]. 

In long-standing type 1 diabetic patients, higher plasma 8-OHdG levels were independently associated with increased risk of DN [177].

### 5.4. Antioxidants

Conflicting results have been reported about total antioxidant status in type 2 diabetic patients; it was reduced [154,157,164,178,179], increased [180], or unchanged [181] compared to controls.

Results from studies examining SOD in type 2 diabetic patients are contradictory, results showed either increased [179,182] or decreased [178,183] SOD activity compared to healthy controls. Studies related to catalase and GSH-Px activity in type 2 diabetic patients compared to healthy controls are also conflicting [105]. Inconsistent results on SOD, catalase, and GSH-Px were also reported in studies comparing type 2 diabetic patients with or without complications [154,155,184,185]. Among patients with complications, patients with DN were also included, but were not analysed separately in these studies. In study by Bondor et al., patients with incipient diabetes-associated nephropathy (defined as estimated glomerular filtration rate (eGFR) < 60 mL/min or urine albumin-to-creatinine ratio (UACR) ≥ 30 mg/g) were included [139]. No difference in SOD activity in patients with or without DN was found [160]. Similar results were shown in other studies [158,161].

No difference in catalase and GSH-Px levels in type 2 diabetic patients with DN compared to those without was found [161,186]. In patients with type 1 diabetes, associations between catalase allelic variations and the prevalence and incidence of DN and ESRD were observed [187].

Haem oxygenases (HOs) are fundamental enzymes in haem catabolism [188]. The HO-1 isoform acts as an antioxidant during oxidative injury [189]. Plasma HO-1 concentrations were significantly increased in newly diagnosed type 2 diabetic patients compared to controls [190]. 

Furthermore, urinary HO-1 levels were significantly increased in diabetic patients with micro- and macroalbuminuria compared to patients with normoalbuminuria and controls [191]. In patients with normoalbuminuria, urinary HO-1 levels were also higher compared to controls [191]. HO-1 was upregulated in lymphocytes in DN patients compared to healthy controls [162].

GSH was decreased in type 2 diabetic patients compared to controls [179,181,183]. Decreased GSH was reported in type 2 diabetic patients with complications (including DN) compared to those without [154]. Furthermore, comparing type 2 diabetic patients with and without DN, plasma GSH was significantly decreased in patients with nephropathy [157]. Interestingly, in the study by Chou et al., no difference in cellular GSH was found in type 2 diabetic patients with and without DN [161]. In the same study, patients with the highest UACR had the lowest levels of vitamin C and vitamin C levels, which correlated negatively with serum creatinine, urine albumin, and UACR [161].

## 6. Antioxidant Therapy

Oxidative stress is involved in the onset and progression of CKD, including DN. Therefore, antioxidant therapy could be an important treatment strategy in these patients. Experimental studies showed beneficial effects of antioxidant therapy in animals [192,193,194,195,196]. Results of antioxidant therapy use in patients with CKD or DN are limited with conflicting results. As shown by a Cochrane database systematic review (including therapy with vitamin E, coenzyme Q, acetylcysteine, bardoxolone methyl, and human recombinant superoxide dismutase), the results of antioxidant therapy have been disappointing in reducing the risk of cardiovascular and all-cause death or major cardiovascular events in CKD patients [197]. However, the authors concluded that current evidence suggests that antioxidant therapy in predialysis CKD patients may prevent progression to ESRD; this finding was based on a very small number of events [197]. Some important review papers have been published in recent years, presenting details of antioxidant therapy for CKD and DN [17,23,43,198,199]. Recently, new medications with antioxidant effects (sacubitril/valsartan, etc.) have come into the spotlight and future clinical trials will determine the efficacy of these or other new drugs in modulating the pro-oxidant milieu of CKD [43,200,201].

## 7. Conclusions

Despite the aggressive blockade of the renin–angiotensin–aldosterone system, many patients with diabetes still progress to ESRD. Oxidative stress is important in the development and progression of DN. A number of pathways and molecules are involved in the induction of oxidative stress in DN. The identification of biomarkers of oxidative stress contributes to our understanding of the development and progression of DN toward ESRD. In this review, we have presented oxidative stress biomarkers used in clinical studies in patients with CKD and DN. To date, these novel biomarkers of oxidative stress cannot replace currently used biomarkers in DN development and progression (eGFR, albuminuria/proteinuria). 

## Figures and Tables

**Table 1:** Most important markers of oxidative stress and antioxidants.

Markers of Oxidative Stress	Antioxidants
***Lipid peroxidation***	***Enzymatic***
Malondialdehyde (MDA)	Superoxide dismutase (SOD)
Thiobarbituric acid reactive substances (TBARSs)	Catalase
4-hydroxynonenal (HNE)	Glutathione peroxidase (GSH-Px)
F2-isoprostanes	Haem oxygenase-1 (HO-1)
***Protein oxidation***	Thioredoxin
Advanced oxidation protein products (AOPPs)	***Nonenzymatic***
Advanced glycation end products (AGEs)	Glutathione (GSH)
Protein carbonyls	Vitamins (vitamins C and E)
***Nucleic acid oxidation***	β-carotene
8-hydroxyguanosine (8-OHG)	
8-hydroxy-2′-deoxyguanosine (8-OHdG)	

**Table 2:** Most important biomarkers of oxidative stress associated with diabetic nephropathy (DN) used in clinical studies.

Biomarker	Clinical Importance	Sample	Ref.
MDA	Increased in patients with DN compared to those without	plasma serum	[152,154] [153]
	No difference in patients with or without DN	plasma	[155]
	No difference in patients with normo-, micro-, and macroalbuminuria	plasma, erythrocytes	[156]
HNE	Increased in patients with DN compared to controls	plasma, leukocytes	[157]
F2-isoprostanes	Increased in patients with DN compared to controls	plasma, leukocytes	[157]
AOPP	Increased in patients with DN compared to those without	serum	[153]
	Increased in patients with DN compared to those without	plasma	[160]
AGE	Increased in patients with DN compared to those without	plasma	[154]
	Increased in patients with DN compared to controls	plasma, urine	[157]
	Increased in patients with renal failure compared to patients with normo-, micro-, and macroalbuminuria without renal failure	blood	[163]
	Increased in haemodialysis patients compared to non-dialysis; no difference in patients with normo-, micro-, and macroalbuminuria	serum	[106]
	No difference in patients with normo-, micro-, and macroalbuminuria	plasma	[156]
Protein carbonyls	Increased in patients with DN compared to those without	serum	[153]
	Increased in patients with DN compared to controls	plasma, leukocytes	[157]
Nucleic acid oxidation	Increased in patients with DN compared to those without	serum urine	[153,167] [167]
	Increased in patients with micro- and macroalbuminuria compared to normoalbuminuria; no difference in patients with micro- and macroalbuminuria	plasma	[156]
	Increased in patients with macroalbuminuria compared to micro- or normoalbuminuria	urine	[106,169]
	Prediction of the onset and progression of DN	urine	[170,171]
	No relationship with the onset and progression of DN	leukocytes	[171]
	No difference in patients with or without DN	urine	[165]
TAS	Decreased in patients with DN compared to those without	plasma	[152]
	No difference in patients with or without DN	plasma	[154]
	No difference in patients with normo-, micro-, and macroalbuminuria	erythrocytes	[156]
SOD	Decreased in patients with DN compared to those without	serum	[153]
	No difference in patients with or without DN	erythrocytes	[155]
	No difference in patients with normo-, micro-, and macroalbuminuria	plasma, erythrocytes	[156]
Catalase	No difference in patients with or without DN	serum erythrocytes	[153] [181]
	No difference in patients with normo-, micro-, and macroalbuminuria	plasma, erythrocytes	[156]
GSH-Px	No difference in patients with or without DN	serum	[153]
	No difference in patients with normo-, micro-, and macroalbuminuria	plasma, erythrocytes	[156]
HO-1	Increased in patients with DN, no difference in patients with micro- and macroalbuminuria	urine	[186]
	Increased in patients with DN compared to controls	lymphocytes	[157]
GSH	Decreased in patients with DN compared to those without	plasma	[152]
	No difference in patients with normo-, micro-, and macroalbuminuria	plasma, erythrocytes	[156]
Vitamin C	Decreased in DN; correlation with UACR and eGFR	tissue	[156]
Vitamin E	No difference in patients with or without DN	tissue plasma	[156] [154]

MDA: malondialdehyde; HNE: 4-hydroxynonenal; AOPP: advanced oxidation protein products; AGE: advanced glycation end products; TAS: total antioxidant status; SOD: superoxide dismutase; GSH-Px: glutathione peroxidase; HO-1: haem oxygenase-1; GSH: glutathione.
